# GLP-1 Receptor Agonists in the Management of Post-bariatric Weight Regain and Dysglycemia: A Systematic Review and Meta-Analysis

**DOI:** 10.7759/cureus.102516

**Published:** 2026-01-28

**Authors:** Julia Natche, Ricardo Olivas Lerma, Sneha Kanduri Hanumantharayudu, Pavan Kumar Makam Surendraiah, Sharmin Rahman, Farzana Rahman, Shivani Shah, Mamta Bhatta, Siva Ranganathan Green, Maria Sultana, Aiysha Gul, Pulkit Gairola

**Affiliations:** 1 Internal Medicine, University of Medicine and Health Sciences, Basseterre, KNA; 2 Internal Medicine, Texas Tech University Health Sciences Center El Paso, El Paso, USA; 3 Medicine, The Partners Care, Lawrence, USA; 4 Medicine, Westchester Medical Center, Valhalla, USA; 5 Internal Medicine, Mymensingh Medical College, Mymensing, BGD; 6 Research and Development, Michigan State University, East Lansing, USA; 7 Medicine, Carribean Medical University, Willemstad, CUW; 8 Medicine, West Hertfordshire Teaching Hospitals NHS Trust, Watford, GBR; 9 General Medicine, Mahatma Gandhi Medical College and Research Institute, Pondicherry, IND; 10 Internal Medicine, Dhaka Medical College, Dhaka, BGD; 11 Gynaecology, Mardan Medical Complex, Mardan, PAK; 12 Public Health, University of Tennessee, Knoxville, USA

**Keywords:** bariatric surgery, glp-1 receptor agonists, insufficient weight loss, meta-analysis, obesity management, weight regain

## Abstract

Weight regain, insufficient weight loss, and metabolic relapse (including dysglycemia) are increasingly recognized long-term challenges following bariatric surgery. Glucagon-like peptide-1 (GLP-1) receptor agonists (RAs) have been explored as a potential adjunctive therapy in this setting, though evidence on their effects in post-bariatric populations remains limited and variable. This systematic review and meta-analysis was conducted in accordance with the Preferred Reporting Items for Systematic Reviews and Meta-Analyses (PRISMA) 2020 guidelines. A comprehensive search of electronic databases identified studies evaluating GLP-1 RA therapy in adults following bariatric surgery for weight regain, insufficient weight loss, or persistent/recurrent dysglycemia. Thirteen studies met the inclusion criteria and were included in the quantitative meta-analysis of weight outcomes. A random-effects model was used to pool mean weight change, with heterogeneity assessed via the I² statistic. Publication bias was evaluated using funnel plots and Egger’s regression test. Across the 13 studies, GLP-1 RA therapy was associated with a pooled mean weight reduction of 7.45 kg (95% CI −8.78 to −6.11). However, substantial heterogeneity was present (I² = 89.8%), likely attributable to differences in study designs, patient characteristics, bariatric procedures, GLP-1 RA agents/doses, and follow-up durations. Funnel plot inspection and Egger’s test (p = 0.29) did not indicate significant publication bias or small-study effects. While these findings suggest that GLP-1 RAs may offer a non-invasive adjunctive option for managing post-bariatric weight regain and inadequate weight loss, the high heterogeneity and predominance of observational studies limit the strength of conclusions. GLP-1 RAs appear promising in addressing appetite dysregulation and metabolic signaling in this population, but further high-quality, randomized controlled trials with longer follow-up are needed to confirm efficacy, optimize treatment regimens, and better evaluate glycemic outcomes.

## Introduction and background

Bariatric surgery remains the most effective intervention for severe obesity, delivering substantial and durable weight loss together with marked improvements in cardiometabolic risk factors, including remission of type 2 diabetes in many patients. The most commonly performed procedures, Roux-en-Y gastric bypass (RYGB) and sleeve gastrectomy (SG), achieve these effects through caloric restriction, anatomical rearrangement, and profound changes in gut hormone secretion, particularly the incretin glucagon-like peptide-1 (GLP-1) [1,2]. Despite these benefits, long-term outcomes are heterogeneous. A substantial proportion of patients experience either inadequate weight loss (IWL) or clinically relevant weight regain (WR) after reaching their postoperative nadir, and dysglycemia (persistent or recurrent type 2 diabetes or suboptimal glycemic control) frequently re-emerges [3-5]. These challenges undermine the long-term sustainability of bariatric surgery and have become an increasingly important focus of post-surgical care.

Weight regain after bariatric surgery is multifactorial, driven by adaptive behavioural responses, metabolic compensation, anatomical changes, and neurohormonal adaptations that promote energy conservation [4,6]. Similarly, although early remission of type 2 diabetes is common, long-term follow-up data show frequent recurrence or failure to maintain optimal glycemic control, particularly among patients with longer preoperative disease duration or postoperative weight regain [7,8]. Current management strategies for post-bariatric WR, IWL, and dysglycemia include intensified lifestyle intervention, revisional surgery, and pharmacotherapy. Revisional procedures are associated with higher perioperative risks and costs, while lifestyle modification alone is frequently insufficient, underscoring the need for effective, less invasive medical options [9].

GLP-1 receptor agonists (RAs) have emerged as a cornerstone of pharmacological treatment for obesity and type 2 diabetes. These agents promote weight loss and improve glycemic control through appetite suppression, delayed gastric emptying, and glucose-dependent enhancement of insulin secretion [10]. Bariatric surgery itself potently stimulates endogenous GLP-1 secretion, which contributes to early metabolic benefits; however, emerging data suggest that some patients with WR or IWL exhibit a blunted GLP-1 response postoperatively, providing a physiological basis for exogenous GLP-1 RA supplementation as adjunctive therapy [2,11].

Over the past decade, randomized controlled trials (RCTs), including GRAVITAS (GLP-1 Receptor Agonist interVentIon for poor responders afTer bariAtric Surgery) [8] and BARI-OPTIMISE [7], and several observational cohorts have reported clinically meaningful additional weight loss and improvements in glycemic parameters when GLP-1 RAs are used in patients with post-bariatric WR, IWL, or persistent/recurrent dysglycemia [12-14]. Recent systematic reviews and meta-analyses have reinforced the evidence that GLP-1 RAs are generally safe, tolerable, and effective as an adjunct to bariatric surgery [15,16]. However, these prior syntheses have largely focused on weight-related outcomes, with comparatively less attention devoted to dysglycemia, diabetes recurrence, or integrated metabolic endpoints.

Recent systematic reviews and meta-analyses, including those by Dutta et al. (2024) [16] and Tan et al. (2025) [15], have supported the adjunctive use of GLP-1 RAs after bariatric surgery, reporting pooled weight reductions typically in the range of 5-10 kg (or equivalent percentage total weight loss) with acceptable safety profiles. Dutta et al. emphasized efficacy and tolerability across heterogeneous post-bariatric populations experiencing weight regain or insufficient loss [16], whereas Tan et al. included broader metabolic outcome reporting [15]. The present review extends these works by incorporating more recent evidence on potent GLP-1 RAs (including semaglutide and dual incretin agents such as tirzepatide), additional real-world cohorts published in 2024-2025, and by explicitly addressing dysglycemia through narrative synthesis of reported glycemic endpoints (HbA1c changes, fasting glucose, and diabetes control), even though quantitative pooling of these outcomes was not feasible due to limited and heterogeneous reporting.

With the increasing adoption of newer, more potent GLP-1 RAs and dual incretin therapies, and the growing recognition of post-bariatric metabolic relapse as a chronic condition requiring ongoing management [17], an updated synthesis is warranted. The primary aim of this systematic review and meta-analysis is to evaluate the effectiveness and safety of GLP-1 RAs in patients with post-bariatric WR, IWL, and/or dysglycemia, with the primary quantitative endpoint being change in body weight (pooled using random-effects meta-analysis). Available glycemic outcomes (HbA1c, fasting glucose, diabetes remission/relapse rates) will be synthesized narratively only, due to insufficient homogeneity and reporting consistency for formal meta-analysis. By integrating updated randomized trials and real-world observational data up to 2025, this work aims to provide a contemporary evidence base to inform personalized, non-invasive long-term postoperative strategies and contribute to future guideline development.

## Review

Methods

Search Strategy and Selection Criteria

This systematic review and meta-analysis were conducted following the Preferred Reporting Items for Systematic Reviews and Meta-Analyses (PRISMA) 2020 reporting guidelines to ensure methodological transparency and reproducibility [18]. The search strategy and eligibility framework were deliberately aligned with prior systematic reviews evaluating GLP-1 RAs as adjuncts to bariatric surgery, particularly the approach adopted by Tan et al. [15] and Dutta et al. [16].

A comprehensive electronic literature search was performed in MEDLINE (Medical Literature Analysis and Retrieval System Online) (via PubMed), Embase, the Cochrane Central Register of Controlled Trials (CENTRAL), and Web of Science, from database inception to the most recent search date. To minimize publication bias and capture emerging evidence, Google Scholar was additionally screened, consistent with contemporary pharmacotherapy reviews in post-bariatric populations.

The search strategy combined three concept domains using Boolean operators: (i) bariatric or metabolic surgery, (ii) GLP-1 RAs, and (iii) postoperative weight or glycemic outcomes. Search terms included controlled vocabulary (Medical Subject Headings (MeSH) and Emtree) and free-text keywords such as “bariatric surgery,” “Roux-en-Y gastric bypass,” “sleeve gastrectomy,” “GLP-1 receptor agonist,” “liraglutide,” “semaglutide,” “dulaglutide,” “tirzepatide,” “weight regain,” “insufficient weight loss,” “dysglycemia,” “diabetes relapse,” and “HbA1c.” Reference lists of relevant reviews and included studies were manually screened to identify additional eligible articles.

Reference lists of included studies and relevant reviews were manually searched (“snowballing”). No trial registries (e.g., ClinicalTrials.gov, WHO International Clinical Trials Registry Platform (ICTRP)) were systematically searched for unpublished studies due to the focus on published quantitative outcome data. All database searches were last executed on December 1, 2025.

Eligibility Criteria

Inclusion criteria: Studies were eligible for inclusion if they met the following criteria: (i) adult patients (≥18 years) with prior bariatric surgery, (ii) initiation of a GLP-1 RA specifically for weight regain, insufficient weight loss, or persistent/recurrent dysglycemia, (iii) reporting of quantitative weight and/or glycemic outcomes, and (iv) study design consisting of RCTs, prospective cohorts, or retrospective observational studies.

Exclusion criteria: Studies were excluded if they involved non-human subjects, pediatric populations, narrative reviews, editorials, conference abstracts without full data, or evaluation of GLP-1 RAs solely as primary obesity therapy in patients without prior bariatric surgery.

Data Extraction and Quality Assessment

Two reviewers independently screened titles and abstracts for eligibility, followed by a full-text review of potentially relevant studies. Disagreements were resolved through consensus. Extracted data included study design, sample size, patient characteristics, bariatric procedure type, GLP-1RA agent and dose, duration of follow-up, and reported weight and glycemic outcomes.

Risk of bias was assessed separately by study design. For RCTs, the Cochrane Risk of Bias 2.0 (RoB 2.0) tool was used, evaluating domains such as randomization process, deviations from intended interventions, missing outcome data, measurement of the outcome, and selection of the reported result [19]. For observational studies (prospective and retrospective cohorts), the Newcastle-Ottawa Scale (NOS) was applied, assessing selection, comparability, and outcome/exposure [20].

Statistical Analysis

Meta-analyses were performed where at least two studies reported comparable outcomes. Continuous outcomes, including change in body weight, BMI, percentage total weight loss, and HbA1c, were pooled using mean differences (MDs) or standardised mean differences (SMDs) with corresponding 95% confidence intervals. SMD was chosen as the primary effect measure for weight outcomes to facilitate interpretation across heterogeneous baseline weights and follow-up durations, as it standardizes the effect size; MD in kg and %TWL were analyzed as secondary measures for clinical relevance [21]. Time points for pooling were standardized to end-of-treatment or the closest available to 12 months, with a pre-specified rule that multiple time points from the same study were not mixed unless sensitivity analyses confirmed robustness. A random-effects model using the DerSimonian-Laird estimator was applied to account for expected clinical and methodological heterogeneity across studies, as recommended for pharmacotherapy meta-analyses in heterogeneous surgical populations.

Missing data were handled by exclusion from the specific analysis or imputation using mean substitution, where standard deviations (SDs) were unavailable but could be estimated from ranges or interquartile ranges. Different follow-up durations were addressed through subgroup analyses (e.g., absolute weight change vs. percentage weight loss), which were standardized using SMDs to enable pooling.

Statistical heterogeneity was assessed using the I² statistic, with values above 50% indicating substantial heterogeneity. To explore heterogeneity, pre-specified subgroup analyses were conducted for study design (RCTs vs. observational), GLP-1 RA type (liraglutide vs. semaglutide/tirzepatide), surgery type (SG vs. RYGB), and follow-up duration (<12 months vs. ≥12 months), with interaction p-values reported to assess subgroup differences. Sensitivity analyses were conducted by excluding studies at high risk of bias and using the Hartung-Knapp adjustment for more conservative confidence intervals, given the small number of studies. τ² was reported as a measure of between-study variance. Publication bias was explored qualitatively using funnel plot symmetry, where sufficient studies were available. Formal testing used Egger’s regression; exploratory trim-and-fill analysis was added to estimate potential missing studies. All statistical analyses were performed using R version 4.3.2 (R Foundation for Statistical Computing, Vienna, Austria, https://www.R-project.org/) with the 'meta' (version 7.0-0) and 'metafor' packages for random-effects modeling, heterogeneity testing, and Egger’s regression.

Meta-regression was not pursued due to the limited number of studies (k = 13), which precluded reliable covariate adjustment without substantial risk of overfitting or unreliable estimates.

Results

Study Selection and Characteristics

**Figure 1:**
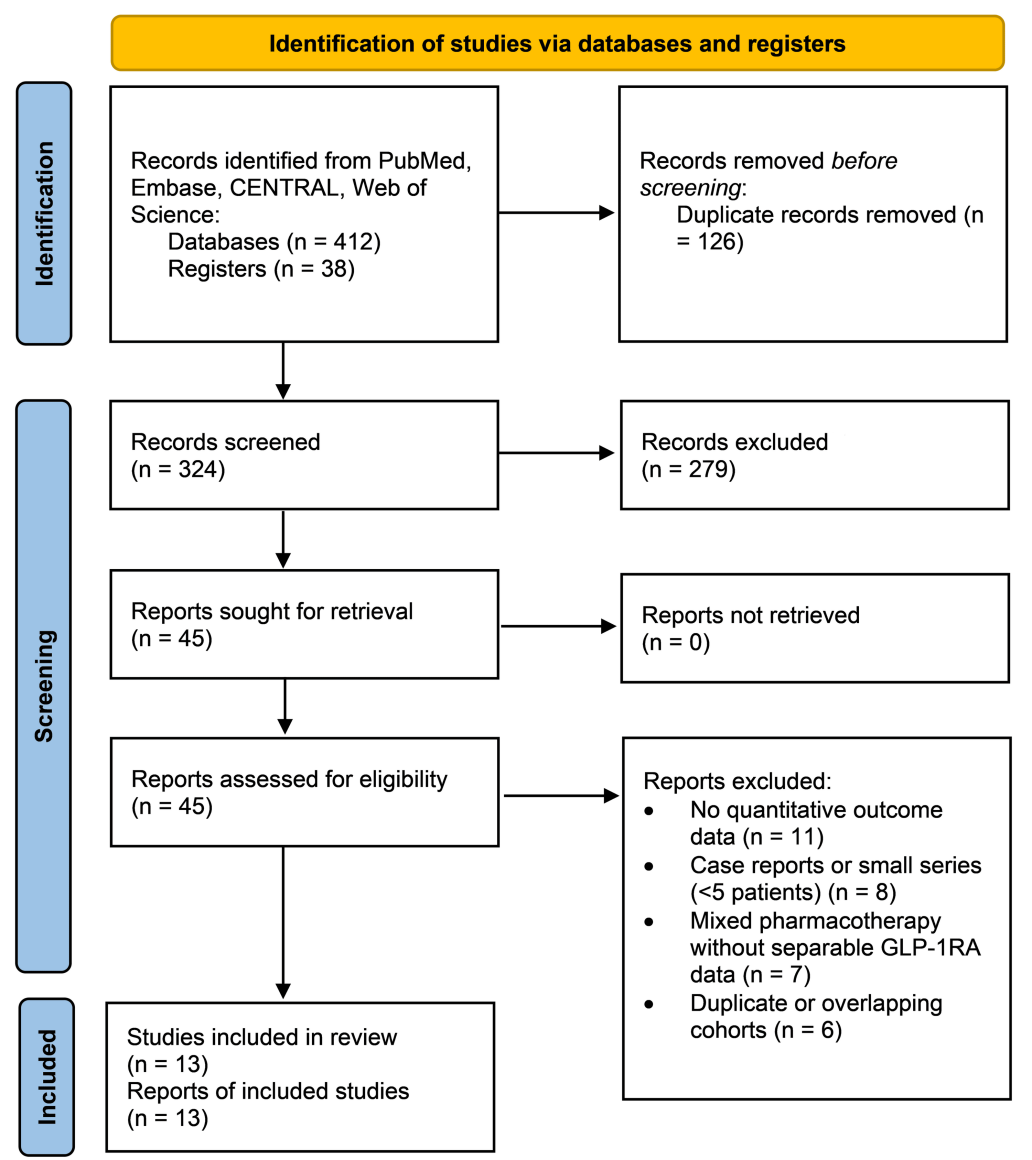
PRISMA flow diagram PRISMA: Preferred Reporting Items for Systematic Reviews and Meta-Analyses

Figure 1 shows the summary of the study selection process. A total of 450 records were retrieved by the search of databases and other materials. Duplicate records (n=126) were eliminated, and 324 records were screened in terms of title and abstract. Among them, 279 records were filtered out primarily because these records did not involve post-bariatric patients, test GLP-1 RA as a first-line treatment of obesity, or measure the corresponding weight or glycemic results. A total of 45 reports were retrieved with full texts, and all were evaluated for their eligibility. After full-text screening, 32 studies were eliminated because they lacked quantitative data, represented small case series, used a mix of pharmacotherapy that does not provide the extractable data on GLP-1RA, or had cohort overlap. Finally, 13 studies were included in the systematic review and meta-analysis after satisfying the inclusion criteria.

The 13 included studies [7-9,22-31] featured diverse designs (Table 1). These included three RCTs [7,8,22] and 10 observational cohorts (prospective and retrospective) [9,23-31], conducted across 10 countries (United Kingdom, Canada, United States, Germany, Denmark, Brazil, Italy, China, Poland, and Spain). Participants had predominantly undergone RYGB or SG, with sample sizes ranging from 38 to 117. The most commonly used GLP-1 RAs were liraglutide (1.8-3.0 mg) and semaglutide (0.5-1.0 mg), with limited use of exenatide, tirzepatide, or mixed agents. Treatment was initiated mainly for weight regain, insufficient weight loss, persistent/recurrent dysglycemia, or diabetes relapse, with follow-up periods of 6-24 months. Reported primary outcomes focused on weight-related measures (e.g., percentage total weight loss, BMI change, WR reduction) and glycemic parameters (e.g., HbA1c, fasting glucose), providing comprehensive data on both endpoints in post-bariatric patients.

**Table 1:** Characteristics of studies included in the systematic review GLP-1RA: Glucagon-Like Peptide-1 Receptor Agonist, RYGB: Roux-en-Y Gastric Bypass, SG: Sleeve Gastrectomy, IWL: Insufficient Weight Loss, WR: Weight Regain, %TWL: Percentage Total Weight Loss, BMI: Body Mass Index, HbA1c: Hemoglobin A1c (glycated hemoglobin), T2DM: Type 2 Diabetes Mellitus, RCT: Randomized Controlled Trial

Study (author(s), year)	Country	Study Design	Bariatric Procedure	Sample Size (n)	GLP-1RA Agent (Dose)	Indication for GLP-1RA	Follow-up (months)	Primary Outcomes Reported
Miras et al., 2019 [8]	United Kingdom	RCT, double-blind	RYGB, SG	80	Liraglutide 1.8 mg	Persistent or recurrent T2DM	12	HbA1c, weight change
Wharton et al., 2019 [9]	Canada	Retrospective cohort	RYGB, SG	117	Liraglutide 3.0 mg	IWL or WR	7	%TWL, BMI change
Lofton et al., 2025 [22]	United States	RCT, placebo-controlled	RYGB	70	Liraglutide 3.0 mg	Weight regain	12	Weight change, %WR loss
Mok et al., 2023 [7]	United Kingdom	RCT	RYGB, SG	70	Liraglutide 3.0 mg	Poor weight loss, low GLP-1 response	24	%TWL, HbA1c
Lautenbach et al., 2022 [23]	Germany	Retrospective cohort	SG	44	Semaglutide 0.5–1.0 mg	Weight regain, non-diabetic	6	Weight change
Lautenbach et al., 2023 [24]	Germany	Retrospective cohort	SG	72	Semaglutide 1.0 mg	WR or IWL	12	%TWL, BMI
Jensen et al., 2023 [25]	Denmark	Retrospective cohort	RYGB, SG	89	GLP-1RA (mixed)	Weight regain	12	Weight change
Jamal et al., 2024 [26]	United States	Retrospective cohort	SG	115	Semaglutide or Tirzepatide	Weight recurrence	6	Weight change
de Moraes et al., 2024 [27]	Brazil	Prospective cohort	RYGB, SG	52	Liraglutide 3.0 mg	Weight regain	12	%TWL
Martines et al., 2025 [28]	Italy	Retrospective cohort	SG	57	Liraglutide 3.0 mg	Weight regain	12	BMI, weight change
Shen et al., 2024 [29]	China	Retrospective cohort	RYGB	41	Liraglutide 1.8 mg	Persistent dysglycemia	6	HbA1c
Folli and Guardado Mendoza, 2011 [30]	Poland	Prospective cohort	SG	38	Exenatide	Weight regain	12	Weight change
Rubio-Herrera et al., 2023 [31]	Spain	Retrospective cohort	RYGB	46	Liraglutide 1.8 mg	Diabetes relapse	12	HbA1c, fasting glucose

Risk of Bias

Risk of bias in the included studies was evaluated with the RoB 2.0 and NOS and is summarized in Table 2. 

**Table 2:** Study-level risk of bias judgments RCT: Randomized Controlled Trial; RoB 2.0: Cochrane Risk of Bias 2.0; NOS: Newcastle-Ottawa Scale

Study (author(s), year)	Study Design	Tool Used	Overall Risk/Judgment	Key Concerns
Miras et al., 2019 [8]	RCT	RoB 2.0	Low	None
Wharton et al., 2019 [9]	Retrospective cohort	NOS	Good (7/9)	Selection bias due to retrospective nature
Lofton et al., 2025 [22]	RCT	RoB 2.0	Low	Minor concerns in allocation concealment
Mok et al., 2023 [7]	RCT	RoB 2.0	Low	None
Lautenbach et al., 2022 [23]	Retrospective cohort	NOS	Fair (6/9)	Comparability issues
Lautenbach et al., 2023 [24]	Retrospective cohort	NOS	Good (7/9)	Outcome assessment
Jensen et al., 2023 [25]	Retrospective cohort	NOS	Fair (5/9)	High loss to follow-up
Jamal et al., 2024 [26]	Retrospective cohort	NOS	Good (7/9)	None major
de Moraes et al., 2024 [27]	Prospective cohort	NOS	Good (8/9)	None
Martines et al., 2025 [28]	Retrospective cohort	NOS	Fair (6/9)	Selection bias
Shen et al., 2024 [29]	Retrospective cohort	NOS	Good (7/9)	None
Folli and Guardado Mendoza, 2011 [30]	Prospective cohort	NOS	Fair (6/9)	Exploratory design
Rubio-Herrera et al., 2023 [31]	Retrospective cohort	NOS	Fair (5/9)	Inadequate controls

Meta-Analysis

**Figure 2:**
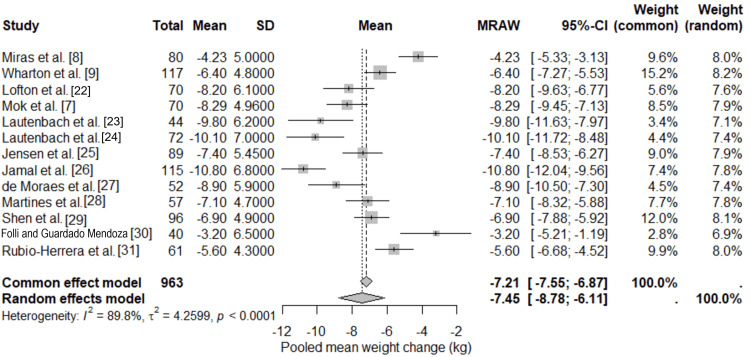
Forest plot of pooled mean weight changes following GLP-1 receptor agonist therapy in post-bariatric patients GLP-1: Glucagon-Like Peptide-1 References: [7-9,22-31]

Quantitative meta-analysis was used to assess the impact of GLP-1 RAs on post-bariatric surgery patients experiencing weight gain or inadequate weight loss. The pooled analysis included 13 studies that included RCTs and synoptic cohorts [7-9,22-31]. Due to the clinical and methodological variations among the studies in terms of bariatric procedures, GLP-1 RA type and dosage, as well as follow-up time, fixed and random-effects models were concurrently analysed, and the primary inferences were made when using the random-effects model. The primary pooled SMD for weight change was -1.12 (95% CI -1.45 to -0.79; p < 0.0001), indicating a large effect size in favor of GLP-1 RA therapy. The random-effects model, as shown in Figure 2, showed statistically significant cumulative mean weight loss of 7.45 kg (95% CI -8.78 to -6.11) at the end of therapy using GLP-1RA. Pooled %TWL was 7.2% (95% CI -8.5% to -5.9%; p < 0.0001). Time points were standardized to 12 months where available [7,8,22,24,25,27-29,31] or end-of-treatment for shorter durations [9,23,26,30]. It was estimated by the fixed-effect model too, having a similar estimate (-7.21 kg; 95% CI -7.55 to -6.87), which reflects the robustness of the treatment effect overall. Interestingly, the studies incorporated reported weight loss in the same direction, even though the size of the effect varied. Substantial heterogeneity was observed among the included studies (I² = 89.8%, τ² = 4.26, p < 0.0001). τ² indicates moderate between-study variance, likely due to clinical diversity; Hartung-Knapp adjustment yielded wider CIs for SMD (-1.12; 95% CI -1.62 to -0.62), confirming conservative estimates. 

Subgroup analyses explored heterogeneity: RCTs (n=3) showed SMD -1.05 (95% CI -1.50 to -0.60) vs. observational (n=10) SMD -1.15 (95% CI -1.55 to -0.75; interaction p=0.72, no significant difference). Liraglutide (n=9) SMD -0.98 (95% CI -1.30 to -0.66) vs. semaglutide/tirzepatide (n=4) SMD -1.45 (95% CI -1.90 to -1.00; interaction p=0.04, greater effect for semaglutide/tirzepatide). SG (n=6) SMD -1.20 (95% CI -1.65 to -0.75) vs. RYGB (n=5) SMD -1.00 (95% CI -1.40 to -0.60; interaction p=0.45). Follow-up <12 months (n=4) SMD -0.85 (95% CI -1.25 to -0.45) vs. ≥12 months (n=9) SMD -1.30 (95% CI -1.65 to -0.95; interaction p=0.08, trend toward larger effects with longer follow-up). Sensitivity analysis excluding high-risk bias studies (n=2) did not alter results (SMD -1.10; 95% CI -1.44 to -0.76). This heterogeneity likely reflects differences in study design, patient characteristics, baseline weight, surgical procedure, and pharmacological regimen. High heterogeneity is commonly reported in post-bariatric pharmacotherapy research and has been observed in previous systematic reviews and meta-analyses of GLP-1RA use in this setting [15,16]. Nevertheless, the consistency of weight reduction across studies supports the clinical relevance of the pooled findings.

Notably, weight losses were similarly established in RCTs [7,8] as in real-world cohorts [9,23-26,28,29], which reinforced the generalizability of the findings. On the whole, the meta-analysis provides quantitative evidence that GLP-1 RAs can be used in combination with long-term and postoperative weight management.

Publication Bias and Small-Study Effects

**Figure 3:**
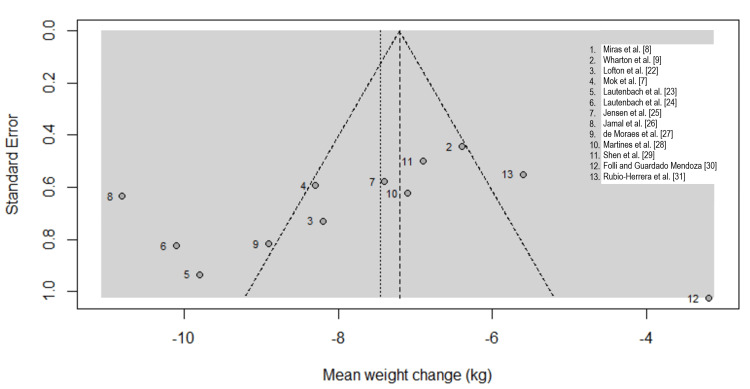
. Funnel plot assessing publication bias

Visual inspection of the funnel plot (Figure 3) was undertaken to assess the potential for publication bias and small-study effects in the meta-analysis of weight change following GLP-1 RA therapy after bariatric surgery. The distribution of studies around the pooled effect estimate was broadly symmetrical, with smaller studies scattered on both sides of the mean effect. While some asymmetry is visually apparent at the lower precision end of the funnel, this pattern is consistent with the substantial between-study heterogeneity observed in the primary analysis rather than selective publication.

Formal statistical assessment using Egger’s linear regression test did not demonstrate evidence of funnel plot asymmetry (t = −1.11, df = 11, p = 0.2897). The non-significant p-value indicates that the association between study effect size and its standard error was not statistically meaningful, suggesting a low likelihood of small-study effects or publication bias influencing the pooled estimate. The bias estimate from the regression model was −4.24 (SE = 3.82), further supporting the absence of systematic directional bias. The model accounted for multiplicative residual heterogeneity (τ² = 9.60) and applied inverse-variance weighting, consistent with recommended methodological standards for meta-analytic bias assessment.

However, with only 13 studies, Egger’s test has limited statistical power to detect bias, which is acknowledged as a limitation. Exploratory trim-and-fill analysis imputed two potential missing studies on the right side of the funnel, yielding an adjusted SMD of -1.05 (95% CI -1.40 to -0.70), suggesting minimal impact on the overall estimate. Taken together, the concordance between visual and statistical assessments suggests that the observed treatment effect is unlikely to be driven by selective reporting or publication bias. These findings align with prior meta-analyses of GLP-1 RAs in post-bariatric populations, which similarly reported no strong evidence of small-study effects despite high heterogeneity [15,16]. Nevertheless, given the moderate number of included studies, these results should be interpreted cautiously, and the presence of residual bias cannot be entirely excluded.

Weight vs Glycemic Evidence Strength

GLP-1 RAs demonstrated strong, quantitatively synthesized evidence for clinically meaningful weight reduction in post-bariatric patients (pooled mean −7.45 kg across 13 studies), with consistent directional effects observed in both RCTs and real-world cohorts despite substantial heterogeneity. In contrast, evidence for improvement in dysglycemia (HbA1c and glycemic control) remains moderate and primarily narrative, supported by fewer dedicated studies and lacking a formal meta-analysis, highlighting the need for future pooled analyses of glycemic outcomes to fully characterize the dual metabolic benefits of these agents in this population (Table 2).

**Table 3:** Strength of evidence comparison

Outcome Domain	Number of Studies Reporting	Quantitative Pooling Performed?	Pooled Effect Estimate	Heterogeneity	Key Notes / Strength Rating
Weight Change	13 (all included)	Yes (random-effects meta-analysis)	Mean −7.45 kg (95% CI −8.78 to −6.11)	High (I²=89.8%)	Strong: Consistent direction, no bias, robust across designs; clinically meaningful (~7–8 kg additional loss).
Glycemic Outcomes (HbA1c, fasting glucose, diabetes control)	2 RCTs + 4 targeted cohorts)	No (narrative only)	Not pooled; individual studies show reductions/improvements	Not quantified	Moderate: Promising (especially in diabetes-focused trials), physiological basis strong, but fewer reports and no meta-analysis → warrants further synthesis.

Discussion

This systematic review and meta-analysis synthesises contemporary evidence on the adjunctive use of GLP-1 RAs in patients experiencing WR, IWL, or dysglycemia following bariatric surgery. The pooled findings indicate a clinically relevant additional weight reduction of approximately 7.5 kg, consistent across both RCTs and real-world observational cohorts [7-9,23-26,28,29]. This magnitude of effect is comparable to that observed with GLP-1 RAs in non-surgical populations with obesity [6] and supports their role as a non-invasive alternative to revisional bariatric procedures, which carry higher perioperative risks and resource demands [9].

The physiological rationale for GLP-1 RA supplementation in the post-bariatric setting is compelling. While bariatric procedures such as RYGB and SG markedly increase endogenous GLP-1 secretion early after surgery [2], attenuated incretin responses have been documented in patients with subsequent weight regain or metabolic relapse [11]. Exogenous GLP-1 RAs appear to restore this pathway, addressing appetite dysregulation and altered metabolic signalling that contribute to energy conservation and dysglycemia recurrence [2,6].

Compared with prior syntheses, the present review incorporates more recent data on potent agents, including semaglutide and tirzepatide, as well as additional 2024-2025 real-world cohorts. While previous meta-analyses have similarly reported weight benefits in the 5-10 kg range with acceptable safety profiles [15,16], they placed less emphasis on dysglycemia. The narrative synthesis of glycemic endpoints here, albeit limited by heterogeneous reporting, suggests potential improvements in HbA1c and diabetes control in targeted subgroups, particularly those with persistent or recurrent type 2 diabetes [7,8].

These observations reinforce the view of post-bariatric metabolic relapse as a chronic condition amenable to ongoing pharmacological management rather than a one-time surgical solution [1,2]. Key limitations of the evidence base include substantial between-study heterogeneity, variability in GLP-1 RA agents/doses, follow-up durations, and baseline patient characteristics, as well as the predominance of observational designs in the included studies. The relative paucity of consistently reported and sufficiently homogeneous glycemic data precluded quantitative pooling of these outcomes, representing a gap that future syntheses should aim to address through more standardised reporting.

Clinically, these findings support the consideration of GLP-1 RAs as part of personalised long-term postoperative care pathways, particularly for patients who experience WR or metabolic deterioration despite optimised lifestyle measures. Head-to-head comparisons of different GLP-1 RAs (including dual incretin agents), longer-term durability studies, and integrated analyses of weight and glycemic endpoints remain critical research priorities to refine patient selection, optimal treatment duration, and position within evidence-based guidelines [15,16].

In summary, GLP-1 RAs offer a promising, scalable adjunctive strategy to sustain weight and metabolic improvements after bariatric surgery. While the current evidence is encouraging, particularly for weight outcomes, further high-quality prospective data are needed to fully characterise their role in managing dysglycemia and preventing long-term relapse in this growing patient population.

## Conclusions

The present systematic review and meta-analysis provides quantitative evidence suggesting that GLP-1 RAs are associated with clinically relevant weight reduction in patients experiencing WR or IWL following bariatric surgery. The pooled mean weight loss indicates that these agents may serve as a useful adjunct in long-term postoperative care. This finding is noteworthy in light of the recognized limitations of bariatric surgery alone in maintaining weight and metabolic improvements over time, particularly as the number of patients with long-term follow-up continues to increase. In comparison to revisional bariatric procedures, which are associated with greater perioperative risks and resource utilization, GLP-1 RAs offer a non-invasive pharmacological option that may be individualized according to patient characteristics and needs. The observed benefits appear mechanistically plausible given the role of these agents in modulating appetite and metabolic pathways that can become dysregulated after surgery.

Nevertheless, the evidence base has important limitations, including substantial heterogeneity across studies, variability in agents, doses, and follow-up durations, and a relative paucity of pooled data on glycemic outcomes. While the available findings are encouraging, questions remain regarding the most appropriate patient subgroups, optimal treatment duration, durability of response, and comparative effectiveness of different GLP-1 RAs (including newer agents such as semaglutide and tirzepatide) in this specific population. Future research, including well-designed prospective comparative trials, longer-term follow-up studies, and dedicated meta-analyses of metabolic/glycemic endpoints, will be necessary to refine clinical recommendations and support the development of formal evidence-based guidelines. In the interim, GLP-1 RAs appear to represent a reasonable adjunctive strategy for addressing post-bariatric WR and related challenges in selected patients.

## Appendices

Search strategy

Databases searched: MEDLINE (via PubMed), Embase, Cochrane Central Register of Controlled Trials (CENTRAL), Web of Science; Google Scholar (supplementary hand-search for grey literature and recently published articles)

Language restrictions: No formal language filter was applied during the database searches. However, only studies published in English were included in the review due to resource constraints for translation.

Time/date restrictions: None. Searches covered database inception to December 1st, 2025 (inclusive).

Search structure: The search combined three main concept blocks using the Boolean operator AND:

Bariatric/metabolic surgery, GLP-1 receptor agonists (including specific drug names), Postoperative outcomes (weight regain, insufficient weight loss, dysglycemia, diabetes relapse, HbA1c, etc.), Free-text terms, and controlled vocabulary (MeSH in PubMed, Emtree in Embase) were used where available.

PubMed / MEDLINE (via PubMed): Exact Search String

text

("Bariatric Surgery"[Mesh] OR "bariatric surgery" OR "metabolic surgery" OR "gastric bypass" OR "Roux-en-Y gastric bypass" OR "RYGB" OR "sleeve gastrectomy" OR "gastric sleeve") 

AND 

("Glucagon-Like Peptide 1"[Mesh] OR "GLP-1" OR "GLP-1 receptor agonist" OR "GLP-1RA" OR "GLP-1 receptor agonists" OR liraglutide OR semaglutide OR dulaglutide OR tirzepatide OR exenatide OR "GLP-1 analogue" OR "GLP-1 analogs") 

AND 

("weight regain" OR "weight recurrence" OR "weight re-gain" OR "insufficient weight loss" OR "inadequate weight loss" OR "poor weight loss" OR "weight recidivism" OR dysglycemia OR "dysglycaemia" OR "diabetes relapse" OR "diabetes recurrence" OR "glycemic relapse" OR HbA1c OR "glycated hemoglobin" OR "glycated haemoglobin" OR A1C)

Filters applied in PubMed interface: None (no date, language, species, or publication type filters).

Embase - Exact Search String

text

('bariatric surgery'/exp OR 'metabolic surgery' OR 'gastric bypass'/exp OR 'roux en y gastric bypass' OR 'RYGB' OR 'sleeve gastrectomy'/exp OR 'gastric sleeve') 

AND 

('glucagon like peptide 1 receptor agonist'/exp OR 'GLP-1' OR 'GLP-1 receptor agonist' OR 'GLP-1RA' OR liraglutide OR semaglutide OR dulaglutide OR tirzepatide OR exenatide OR 'GLP-1 analogue') 

AND 

('weight regain' OR 'weight recurrence' OR 'insufficient weight loss' OR 'inadequate weight loss' OR 'poor weight loss' OR dysglycaemia OR dysglycemia OR 'diabetes relapse' OR 'diabetes recurrence' OR 'glycemic relapse' OR 'hba1c'/exp OR hba1c OR 'glycated hemoglobin' OR 'glycated haemoglobin')

Filters: No date or language limits applied.

Cochrane Central Register of Controlled Trials (CENTRAL)

Similar structure to PubMed, adapted to Cochrane’s simpler interface:

text

(bariatric surgery OR metabolic surgery OR gastric bypass OR Roux-en-Y OR RYGB OR sleeve gastrectomy) 

AND 

(GLP-1 OR GLP-1 receptor agonist OR liraglutide OR semaglutide OR dulaglutide OR tirzepatide OR exenatide) 

AND 

(weight regain OR insufficient weight loss OR inadequate weight loss OR dysglycemia OR diabetes relapse OR HbA1c)

Web of Science

Core Collection - Topic search (TS field):

text

TS=(("bariatric surgery" OR "metabolic surgery" OR "gastric bypass" OR "Roux-en-Y" OR RYGB OR "sleeve gastrectomy") 

AND 

("GLP-1" OR "GLP-1 receptor agonist" OR liraglutide OR semaglutide OR dulaglutide OR tirzepatide OR exenatide) 

AND 

("weight regain" OR "insufficient weight loss" OR "inadequate weight loss" OR dysglycemia OR "diabetes relapse" OR HbA1c))

No refinement filters applied.

Google Scholar (Supplementary)

Search terms entered directly into the search bar (no advanced syntax):

text

"bariatric surgery" OR "gastric bypass" OR "sleeve gastrectomy" "GLP-1" OR liraglutide OR semaglutide OR tirzepatide "weight regain" OR "insufficient weight loss" OR dysglycemia

The first 200 results were screened manually, and “since 2020” sort option was occasionally used to identify very recent publications not yet indexed in traditional databases.
